# Supplementary material for: Cleavage-intermediate Lassa virus trimer elicits neutralizing responses, identifies neutralizing nanobodies, and reveals an apex-situated site-of-vulnerability
Source: Nat Commun. 2024 Jan 4;15:285. doi: 10.1038/s41467-023-44534-y (PMC10767048; doi:10.1038/s41467-023-44534-y)
Supplement: Supplementary file 1 — Supplementary Information [file 41467_2023_44534_MOESM1_ESM.pdf]

Construct description	Physiological	Lower pH (5.5)	Higher temp (56° C)
Lassa_50A10THS	1.4936	3.0184	1.047
Lassa_50A15THS	1.4354	3.0665	0.9952
Lassa_50A12THS	0.9944	2.3964	0.6915
Lassa_R325CM359C_deloriDS_	0.501	0.7142	0.3089
Lassa_R207GC_E239P_4R_G360C_T331P	0.3274	0.3487	0.2654
Lassa_FdTHS	0.3234	0.6245	0.1757
Lassa_DS1	0.3108	0.2583	0.1827
Lassa_R207GC_E239P_4R_G360C_A343G	0.2984	0.2968	0.1912
Lassa_R207GC_E239P_4R_G360C_S332G	0.2873	0.3602	0.1783
Lassa_R207GC_E239P_4R_G360C_S332P	0.2307	0.2678	0.1485
**LASSA_5VK2**	0.2287	0.1834	0.1508
Lassa_R207GC_E239P_4R_G360C_T331G	0.2285	0.2764	0.1708
Lassa_303Y	0.2241	0.2088	0.1486
Lassa_R207GC_E239P_4R_G360C_N342G	0.2237	0.2346	0.1776
Lassa_248W	0.2229	0.2029	0.1504
Lassa_R207GC_E239P_4R_G360C_Q330G	0.216	0.2095	0.1491
Lassa_R207GC_E239P_4R_G360C_Q330P	0.2142	0.2286	0.1609
Lassa_bRFdTHS	0.2055	0.2751	0.1181
Lassa_R207GC_E239P_4R_G360C_K327G	0.1892	0.1963	0.1461
Lassa_R207GC_E239P_4R_G360C_K327P	0.1628	0.1784	0.1214
Lassa_305W	0.1587	0.1298	0.102
Lassa_303W	0.1542	0.1713	0.143
LassaGP_144C147C	0.1524	0.1539	0.1115
LassaGP_81C319C	0.1518	0.1253	0.1337
Lassa_R207GC_E239P_4R_G360C_V341G	0.1517	0.1312	0.0987
Lassa_305F	0.1344	0.1516	0.1118
LassaGP_87C198C	0.1274	0.1107	0.1078
Lassa_R193M_Q247M_K339M	0.1271	0.1199	0.107
Lassa_R193M_D211M_Q247M	0.1221	0.0983	0.0969
Lassa_Q247M_K339M	0.116	0.1341	0.0982
Lassa_R193M_D211M_K339M	0.1102	0.1034	0.0967
Lassa_R207GC_E239P_4R_G360C_N346G	0.106	0.1248	0.0953
LassaGP_113C165C	0.1056	0.1165	0.1005
Lassa_D211M_Q247M_K339M	0.1033	0.0996	0.1093
Lassa_R193M_D211M_Q247M_K339M	0.1012	0.0909	0.0894
LassaGP_246C347C	0.0993	0.087	0.0954
LassaGP_356C361C	0.0966	0.0917	0.086
LassaGP_196C240C	0.0961	0.105	0.123
LassaGP_168C184C	0.096	0.0739	0.0732
LassaGP_348C343C	0.096	0.0904	0.1082
Lassa_M73CI286C	0.0933	0.0926	0.1343
Lassa_DiSu1_R193M_D211M_Q247M_R250F_K339M	0.0915	0.0958	0.1004
LassaGP_1	0.0914	0.0859	0.0884
LassaGP_197C234C	0.0912	0.0803	0.0919
LassaGP_2	0.0905	0.0788	0.0907
Lassa_L326CG208GC	0.0901	0.0894	0.0912
LassaGP_348C260C	0.0896	0.0732	0.0815
Lassa_403W	0.0895	0.1111	0.0878
**LASSA WT**	0.0894	0.1118	0.0896
Lassa_R193M_D211M_Q247M_R250F_K339M	0.0886	0.0864	0.0914
Lassa_R207GC_E239P_4R_G360C_L344G	0.0882	0.0759	0.0816
LassaGP_3	0.0871	0.0829	0.1431
LassaGP_107C217C	0.085	0.0938	0.1188
Lassa_I345P	0.0841	0.0764	0.0809
Lassa_R207GC_E239P_4R_G360C_I345G	0.0817	0.0904	0.0943
Lassa_N74CL285C	0.0812	0.0824	0.0846
LassaGP_85C240C	0.0812	0.0722	0.0794
Lassa_T263CA343C	0.0809	0.073	0.0867
LassaGP_143C244C	0.0807	0.0742	0.0792
LassaGP_198C234C	0.0805	0.0771	0.0823
Lassa_396W	0.0803	0.0819	0.0844
LassaGP_62C407C	0.0787	0.0642	0.0744
Lassa_407W	0.0786	0.0861	0.0778
LassaGP_353C363C	0.078	0.0804	0.0772
**LASSA GP2**	0.0769	0.0685	0.0811
Lassa_Q69CY371CR325CM359C	0.0764	0.0645	0.0758
Lassa_N74CM284C	0.0759	0.07	0.0773
LassaGP_314C344C	0.0759	0.0675	0.0814
LassaGP_311C345C	0.0748	0.07	0.077
Lassa_DiSu2_R193M_D211M_Q247M_R250F_K339M	0.0741	0.0818	0.0847
Lassa_DiSu3_R193M_D211M_Q247M_R250F_K339M	0.0738	0.0689	0.0808
**no DNA**	0.0736	0.0662	0.0772
Lassa_DS4	0.0729	0.0673	0.0881
Lassa_DS2	0.0727	0.0682	0.0799
LassaGP_369C383C	0.0723	0.0723	0.0759
LassaGP_362C389C	0.072	0.0704	0.075
Lassa_DiSu1_2_R193M_D211M_Q247M_R250F_K339M	0.0719	0.0665	0.0808
LassaGP_145C251C	0.0719	0.0656	0.0733
LassaGP_347C260C	0.0718	0.064	0.1002
**no DNA**	0.0717	0.0647	0.0727
Lassa_DiSu1_3_R193M_D211M_Q247M_R250F_K339M	0.0712	0.0664	0.0795
Lassa_DiSu1_2_3_R193M_D211M_Q247M_R250F_K339M	0.0712	0.0667	0.0778
LassaGP_356C363C	0.0707	0.0635	0.0731
LassaGP_368C386C	0.0705	0.0649	0.0859
Lassa_DS3	0.0705	0.0691	0.0792
Lassa_E392CG98C	0.0701	0.0649	0.0774
LassaGP_166C220C	0.0699	0.0634	0.0778
**LASSA GP1**	0.0683	0.0626	0.0734

Control wells

Top hits from initial ELISA

Top hits after 1L expression and characterization

Construct description	Physiological	Lower pH (5.5)	Higher temp (56° C)
Lassa_FD2	0.4772	1.002	0.4313
Lassa_D347P	0.4744	0.6894	0.396
Lassa_FD3	0.4055	0.8205	0.3533
Lassa_FD2F305	0.3608	0.74	0.263
Lassa_FD4	0.3357	0.5328	0.2846
Lassa_FD1	0.2582	0.5977	0.2527
**LASSA_5VK2**	0.2119	0.2572	0.1772
Lassa_MF1	0.2079	0.27	0.1932
Lassa_MF3	0.1626	0.2868	0.1779
Lassa_FD2W303	0.1515	0.2317	0.1536
Lassa_R193M	0.147	0.1659	0.1182
Lassa_R193K339E	0.1462	0.1643	0.134
Lassa_K339M	0.1424	0.1397	0.1163
Lassa_MF2	0.1334	0.2285	0.1421
Lassa_Q247M	0.1314	0.1668	0.1181
Lassa_1nza	0.1296	0.2481	0.1659
Lassa_R250F	0.1289	0.1633	0.1239
Lassa_R193K339D	0.122	0.1562	0.1151
Lassa_R193M_K339M	0.1218	0.1346	0.1114
LassaSS4	0.1205	0.1081	0.1051
Lassa_R193M_Q247M	0.1169	0.1336	0.1109
LassaSS5	0.1159	0.0796	0.0846
Lassa_MF4	0.1156	0.1656	0.1306
Lassa_D211M_Q247M	0.1093	0.1321	0.1119
Lassa_FD2C72C368	0.1072	0.0887	0.0885
Lassa_D211M	0.1066	0.1208	0.1018
Lassa_Q189LR193L_M351F	0.105	0.1137	0.0984
Lassa_b_v6	0.1027	0.1101	0.0848
Lassa_MF4C67C373	0.1026	0.0862	0.0805
Lassa_N346P	0.0989	0.0682	0.0799
Lassa_342F_N346FV388L	0.0975	0.1026	0.0952
Lassa_R193M_D211M	0.0958	0.1003	0.0964
Lassa_D211M_K339M	0.0926	0.0968	0.0886
LassaSS8	0.0898	0.0909	0.0959
Lassa_Disu1_3	0.0881	0.0689	0.0777
Lassa_DiSu3	0.086	0.0913	0.1078
Lassa_C263C352P130	0.0849	0.0753	0.103
**LASSA WT**	0.0823	0.0809	0.0771
Lassa_MF2sc1	0.0799	0.1071	0.0875
Lassa_Disu1_2_3	0.0794	0.0771	0.0802
Lassa_FD3sc1	0.0779	0.1014	0.0894
LassaSS6	0.0767	0.0861	0.0901
Lassa_D306FD302F	0.0766	0.0753	0.0782
Lassa_C138C254	0.0765	0.0768	0.0946
**LASSA GP1**	0.0762	0.0659	0.0768
Lassa_MF4C72C368	0.0757	0.0844	0.0863
Lassa_T70CW370C	0.0753	0.079	0.0881
Lassa_MF3sc1	0.0747	0.0771	0.0807
Lassa_C263C352_C138C254	0.0746	0.0701	0.0836
Lassa_b_v5	0.0746	0.0801	0.089
Lassa_L71CY369C	0.0738	0.1221	0.0889
Lassa_FD2sc1	0.0736	0.085	0.0887
Lassa_C263C352sc1	0.0732	0.0798	0.0866
Lassa_turn329v2	0.0725	0.0768	0.0806
Lassa_FD2sc3	0.0722	0.0716	0.0853
Lassa_C263C348	0.0722	0.0735	0.0841
Lassa_C263C352sc2	0.072	0.0766	0.0787
Lassa_turn329v3	0.0717	0.0809	0.0811
Lassa_turn170_v1	0.0712	0.0677	0.0768
Lassa_342F_N346F	0.0712	0.0658	0.0788
Lassa_FD2C343C348	0.0708	0.0883	0.0834
Lassa_C263C352P131	0.0707	0.069	0.0774
Lassa_D306LD302L	0.0706	0.0735	0.0749
**LASSA GP2**	0.0706	0.0708	0.0942
Lassa_FD2C67C373	0.0704	0.0675	0.0785
Lassa_turn170_v2	0.0702	0.0687	0.0801
Lassa_b_v7	0.0702	0.066	0.0761
Lassa_b_v2	0.07	0.0679	0.0758
Lassa_C263C349	0.0698	0.0691	0.0859
Lassa_Disu2_3	0.0698	0.0694	0.0776
Lassa_FD2C263C352sc1	0.0696	0.0698	0.0823
Lassa_MF4C263C352sc1	0.0696	0.0671	0.0782
Lassa_385s	0.0694	0.0716	0.0776
Lassa_FD2sc2	0.0693	0.0702	0.0845
Lassa_DSS1E72CK368CQ69CY371C	0.0692	0.0653	0.0761
**no DNA**	0.0689	0.0654	0.073
Lassa_b_v4	0.0688	0.0679	0.074
Lassa_b_v1	0.0686	0.0652	0.0756
Lassa_E72CK368C	0.0684	0.0695	0.085
Lassa_MF4C263C352	0.0683	0.0673	0.0838
Lassa_DiSu1_2	0.0682	0.0799	0.0782
Lassa_b_v3	0.0681	0.0667	0.0768
Lassa_I410E_M414E	0.068	0.0644	0.0752
Lassa_Q69CY371C	0.0678	0.0689	0.0769
**no DNA**	0.0678	0.0679	0.074
Lassa_turn329v4	0.0676	0.0669	0.0767
Lassa_FD2C263C352	0.067	0.0675	0.0783
Lassa_C263C352C67C373	0.0658	0.0657	0.0735

Strong 37.7 H binding

Medium 37.7 H binding

Weak 37.7H binding

Supplementary Fig. 1 | Antigenic screening of designed prefusion-stabilized Lassa GPC constructs.
We designed 164 variants, which we screened for binding to the quaternary-specific GPC antibody 37.7H under physiological conditions, at lower pH (5.5) and at higher temperature (56° C). Numbers indicate ELISA results (OD_{450}) for recognition by 37.7H. Top hits for each of the two plates are highlighted in red fonts. Controls are indicated with purple background. Highlighted in blue in the "Construct description" column are the two designs, inter-protomer disulfide and foldon fusion, that were combined in the final construct for detailed characterization in this study after evaluation of their behavior at 1 liter expression and the ability to combine them.

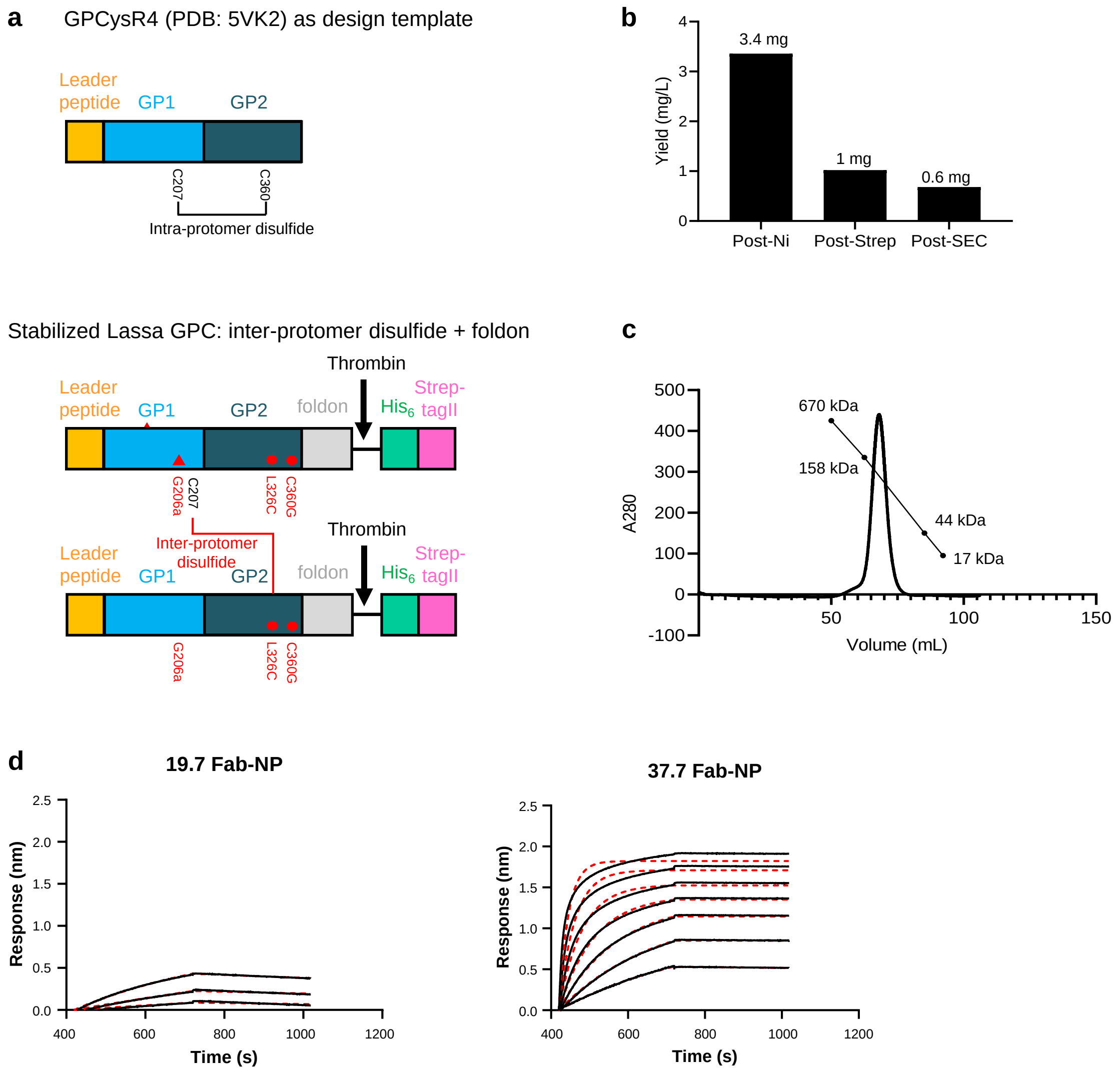

mAbs	K_D (M)	K_D error	k_{on} (1/Ms)	k_{on} error	k_{off} (1/s)	k_{off} error
19.7	4.16E-10	2.86E-09	2.00E+04	6.02E+02	3.19E-05	<1.0E-07
37.7	<1.0E-12	2.69E-10	8.11E+04	1.75E+03	<1.0E-07	

Supplementary Fig. 2 | Construct, yield, and purification profile of stabilized soluble Lassa GPC trimer.

a. Schematic showing the design of GPCysR4 (top) and stabilized soluble Lassa GPC trimer (below). The original C207-C360 intra-protomer disulfide in GPCysR4 was abolished by mutating C360G. New inter-protomer disulfide was created between C207 and L326C. Insertion of G206a allowed optimal geometry for such disulfide bond formation. T4-fibritin (foldon) trimerization domain was also introduced at the C-terminus to fix the base of the trimer.

b. Protein yield of the stabilized soluble GPC trimer following nickel-affinity (Ni), streptavidin-affinity (Strep), and size exclusion (SEC) purification.

c. SEC profile of the stabilized soluble GPC trimer on Superdex 200 16/600 column.

d. Antigenicity of GPC trimer Enc nanoparticle measured by BLI binding of human Lassa nAbs.

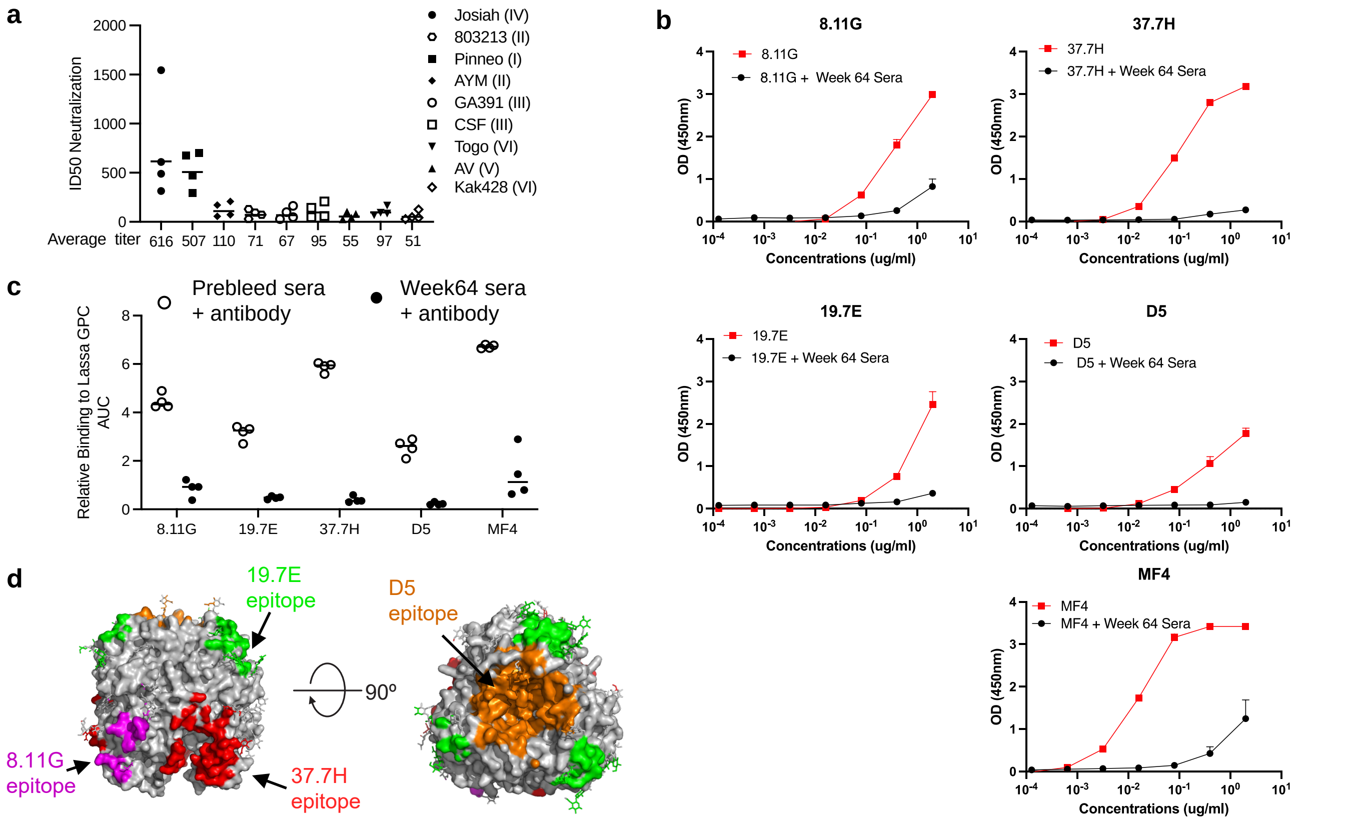

e Rhesus macaque immunization

Group	Name	# Animals	Immunization (week 0, 4, 8, 12, 16, 20)
1	LASV GPC trimer	5	GPC trimer
2	LASV GPC trimer nanoparticle	5	GPC trimer nanoparticle

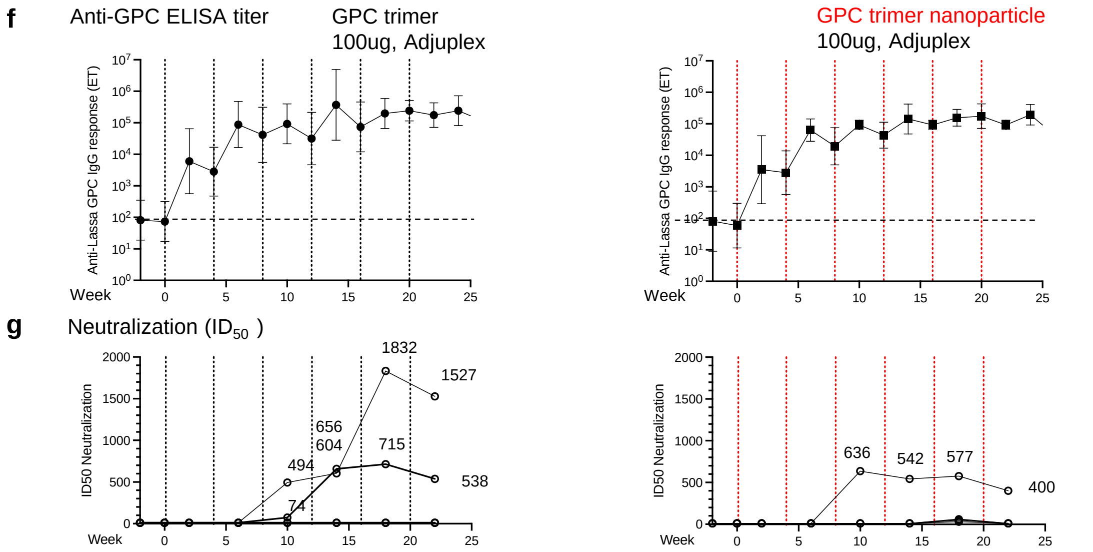

Supplementary Fig. 3 | Sera analysis demonstrate that elicited neutralizing serum responses compete with apex binder D5 as well as other GPC-trimer binding antibodies.

a. Cross neutralizing responses elicited after GPC trimer prime and nanoparticle boost at week64.

b. Sera at week64 can block neutralizing antibodies binding to GPC trimer. Assay was done by adding post-immunization Guinea pig sera first and then adding 2ug/ml Ab to see if binding of Ab to binding site was blocked by antibodies in the sera.

c. Immunized guinea pigs show blocking of all antibodies binding to GPC trimer compared to prebleed.

d. The location of the epitopes on the GPC trimer, 19.7E epitope (Perrett et al., 2023, Cell Rep 42, 112524) shown as green, 8.11G epitope shown as magenta, 37.7H epitope shown as red and D5 epitope shown as brown.

e. Rhesus macaque immunization schedule.

f. Immune responses against to Lassa GPC trimer were measured by ELISA. The initial dilution show as dotted line.

g. Both Lassa GPC trimer and nanoparticle immunizations can elicit neutralizing responses in Rhesus macaque.

a

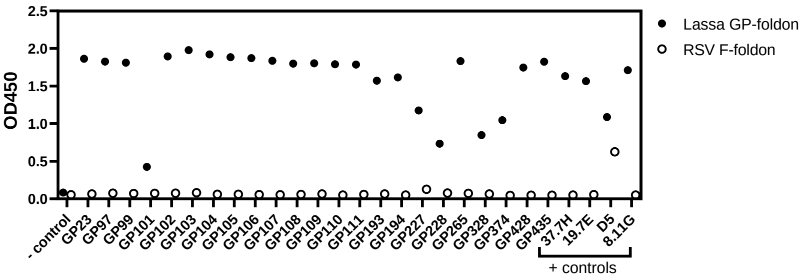

b

Ab ID	Ab full name	VH	VH SHM	JH	CDRH3 length	CDRH3 sequence	VK/VL	VK/VL SHM	JK/JL	CDRL3
GP23	GP2011-5.098.1	VH1-68-54	17.2	JH1-54	20	CARDRRYYSGSGGVYYFEIW	VK1-14-24	13.8	JK1-37	CQQDQTWPPTF
GP97	GP2011-2.001.1	VH3-157-75	9.5	JH1-54	18	CVRDSFWVGTPHPYFDIW	VK1-242-37	3.8	JK2-37	CQQHNNLPLTF
GP99	GP2011-2.001.7	VH3-157-75	11.9	JH1-54	18	CARGTIWVGTPHPYFDIW	VK1-242-37	4.3	JK2-37	CQQHNNLPLTF
GP101	GP2011-2.001.3	VH3-157-75	12.9	JH1-54	18	CARGTIWVGTPHPYFDIW	VK1-242-37	4.7	JK2-37	CQQHNNLPLTF
GP102	GP2011-2.002.2	VH3-157-75	10.9	JH1-54	18	CARGTFWVGTPHPFFDIW	VK1-242-37	5	JK2-37	CQQHANLPFTF
GP103	GP2011-2.001.6	VH3-157-75	8.5	JH1-54	18	CARGTIWVGTSHPHFDMW	VK1-242-37	5.7	JK2-37	CQQHNNLPFTF
GP104	GP2011-2.001.8	VH3-157-75	8.5	JH1-54	18	CARGTIWVGTSHPYFDAW	VK1-242-37	6.6	JK2-37	CQQHNNLPFTF
GP105	GP2011-2.047.1	VH3-157-75	7.8	JH1-54	18	CARGTIWYGTSHPYFDIW	VK1-242-37	6.9	JK2-37	CQQHGSLPFTF
GP106	GP2011-2.001.4	VH3-157-75	11.2	JH1-54	18	CARGTIWVGTSHPYFDSW	VK1-242-37	7	JK2-37	CQQHGNLPFTF
GP107	GP2011-2.001.5	VH3-157-75	10.9	JH1-54	18	CAKGTIWVGTPHPYFDVW	VK1-242-37	7.3	JK2-37	CQQHNNFPFTF
GP108	GP2011-2.001.2	VH3-157-75	9.5	JH1-54	18	CARGTIWFGTPHPYLDIW	VK1-242-37	7.8	JK2-37	CQQHGNLPFTF
GP109	GP2011-2.001.9	VH3-157-75	13.9	JH1-54	18	CARGTIWVGTSHPHFDVW	VK1-242-37	8	JK2-37	CQQHNNLPFTF
GP110	GP2011-3.042.1	VH3-157-75	9.5	JH1-54	17	CVRDVHYVGGLPSFDVW	VK1-242-37	8.8	JK2-37	CQQHHKLPLTF
GP111	GP2011-2.002.1	VH3-157-75	11.9	JH1-54	18	CTRGTIWFGTSHPYFDVW	VK1-242-37	11.5	JK2-37	CQQHKNLPFTF
GP193	GP2011-2.084.1	VH3-230-54	11.2	JH2-54	9	CAGGPLQYW	VK2-16-37	5.4	JK2-37	CVQTTQYPYTF
GP194	GP2011-2.083.1	VH3-183-54	11.1	JH5-54	13	CVRADDPYAFDIW	VK2-16-37	9	JK2-37	CVQTTHDPLTF
GP227	GP2011-3.066.1	VH1-91-54	6.5	JH1-54	18	CARETFSSGFLYSFFDIW	VK3-170-37	5.3	JK3-37	CYQYSHGGTF
GP228	GP2011-3.076.1	VH1-91-54	6.9	JH1-54	18	CTRETFSSGFTYSYFDVW	VK3-170-37	5.3	JK3-37	CYQYSHGGTF
GP265	GP2011-3.044.1	VH3-185-54	10.2	JH1-54	11	CASGTYNFDIW	VK4-77-37	5.3	JK1-37	CQQSVNAPPWTF
GP328	GP2011-3.075.1	VH3-287-54	2.1	JH1-54	18	CATTGGSTNYGDYYFDVW	VL3-32-4	10	JL10-4	CQVWDSDSAHYVF
GP374	GP2011-3.047.1	VH1-115-54	4.2	JH5-54	16	CSRSGGGWGYFPLDIW	VL4-121-4	8	JL10-4	CGSNYNIGGSFGWVF
GP428	GP2011-5.027.1	VH3-197-54	10.2	JH1-54	14	CAKGRQYVSLFDVW	VL8-104-4	3.6	JL10-4	CGADDGTGTRLVYVF
GP435	GP2011-3.005.1	VH3-197-54	7.8	JH1-54	14	CVKGRQYVGELDVW	VL8-87-4	7.2	JL10-4	CGADYSDESSLQYVF

Ab ID	Heavy Chain	Light Chain
GP23	QVQLQESGPGLVKPSQALSLTCSVSGFPITTLYYGWHWIRQPKGKSPEWMGVINYKGETTYNPSLQ<br>SRISISRETGKNQFFLHLNSVTDEDTAIYYCARDRRYYSGSGGVYYFEIWGVGTLVTVSS	DIQLTQPASVSASVGDTVTISCWASQSVTSSLQWYQQQPGHAPQLLVYRATNSPPGVPSRFR<br>GSGSGTHFVLTINNLQPEDFAMYYCQQDQTWPPTFGAGTKLEIK
GP97	EEQLVESGGDLVQPGGSLRLSCVASGFTFSDYYMSWIRQAPGKGLEWISRITNNNNAPRYAGSVRD<br>RFTISRDNGKTTLYLQMSSLRPEDTAMYYCVRDSFWVGTPHPYFDIWGAGTLVTVSS	DVQLTQPPSASASVGDTVKISCRASQSVGSYLNWYQQKPGQPPKPLIYHATNLQSGVPSRFS<br>GSGSGTDFTLTITGLQPEDFTTYYCQQHNNLPLTFGAGTKVEIK
GP99	EDQLVESGGDLVQPGGSLRLSCVASGFTFSNYFMNWVRQAPGKGLEWISRIGDNGRAARYTDSVQG<br>RFTISRDNDKNTLYLQMSSLRPEDTAVYYCARGTIWVGTPHPYFDIWGAGTLVTVSS	DVQLTQPPSASASVGDTVKINCRASQSVGSYLNWYQQKSGQAPKPLIYHATNLQFGVPSRFS<br>GSGSGTDFTLTISGLQPEDFATYYCQQHNNLPLTFGAGTKLDVK
GP101	EDQLVESGGGLVQPGGSLRLSCVASGFTFSNYFMNWVRQAPGKGLEWISRVGDNGRAARYTDSVQG<br>RFTISRDNDKNTLFLQMTSLRPEDTAVYYCARGTIWVGTPHPYFDIWGAGLVTVSS	DVQLTQPPSASASVGDTVKINCRASQSVGSYLNWYQQKSGQPPKPLIYHATNLQFGVPSRFS<br>GSGSGTDFTLTISGLQPEDFATYYCQQHNNLPLTFGAGTKLDVK
GP102	EDYLVESGGGLVQPGGSLRLSCVASGFTFSDHYMNWIRQAPGKGLEWISRVGHNGLSPKYIDSVKG<br>RFTISRDNGKNTLYLQLTNVRPEDTAVYYCARGTFWVGTPHPFFDIWGAGTLVTVSS	DVQLTQPPSASASVGDTVKISCRASQSVRTYLNWYQQKPGQPPKPLIYGGTNLHSGVASRFS<br>GSGSGTDFTLTISGLQPEDFATYYCQQHANLPFTFGAGTRMEIK
GP103	EDQLVESGGGLVQPGGSLRLSCVASAFTFSDYYMNWIRQAPGKGLEWISRIGHNGGASKYADPVKG<br>RFTISRDNDKSTLYLQMNSLGPEDTAVYYCARGTIWVGTSHPHFDMWGAGTLVTVSS	DIQLTQPPSASGSVGDTVKISCRASQSIRTYLNWYQQKPGQTPKPLIYGATNLEFGVPSRFS<br>GSGFGTDFTLTISGLQPEDFATYYCQQHNNLPFTFGAGTKLEMR
GP104	EEQLVESGGGLVQPGGSLRLSCVASGFTFSDHYMNWIRQAPGKGLEWISRIGHNGRAEKYTDSVEG<br>RFTISRDNDRNTLYLQMSSLRPEDTAVYYCARGTIWVGTSHPYFDAWGAGTLVTVSS	DVQLTQPPSTSASVGDTVKISCRASQSVGRYLNWYQQEPGQAPKPLIYGATNLRAGVPSRFS<br>GSGSGTDFTLTISGLQPEDFTTYYCQQHNNLPFTFGAGTKVEIK
GP105	EEQLVESGGGLVQPGGSLRLSCVASGFTFTNYYMSWIRQTPGKGLEWISRISNEGSDTKYTDFVKG<br>RLTISRDNGKNTLYLQMSSLRPEDTAVYYCARGTIWYGTSHPYFDIWGAGTLVTVSS	DIQLTQPPSVSASVGDTVKLSCRASQSPRNYLNWYQQKSGQAPKPLISYATNLLSGVPSRFS<br>GSGSGTDFTLTISGLQPEDFATYYCQQHGSLPFTFGAGTKLEIR
GP106	EEQLIESGGGLVQPGGSLRLSCVASGFTFSDHYMNWIRQPPGKGLEWISRVGHNGRAEKYADSVKG<br>RFTISRDNGKNTLYLQMSSLRPDDTGVYYCARGTIWVGTSHPYFDSWGAGTLVTVSS	DVQLTQPPSASASVGDTVKITCRASQSVRTYLNWYLQEPGQAPKSLIYGATNLQAGVPSRFS<br>GTGSGTDFTLTITGLQPEDFATYYCQQHGNLPFTFGAGTRLEIK
GP107	EDQLVESGGDLVQSGGSLRLSCAASGFTFNDYYMNWIRQAPGKGLEWVSRIGHNGRAEKYVDSVKG<br>RFTISRDNGKNILYLQMTGLRPEDTAVYYCAKGTIWVGTPHPYFDVWGTGTLVTVSS	DVHLTQPPSASASVGDTVKISCRASQSVGRYINWYQQRPGQAPKPLIFGATNLHFGVPSRFT<br>ASGSGTDFTLTISGLQPEDFATYYCQQHNNFPFTFGAGTKLEIK
GP108	EDQLVESGGGLVQPGGSLRLSCVASGFTFSNYFMNWVRQAPGRGLEWISRIGNNGNSPRYADFVKG<br>RLTISRDNDKNTLYLQINNLRPEDTAVYYCARGTIWFGTPHPYLDIWGAGTLVTVSS	DIQLTQPPSASASVGDTVKLSCRASQSLGTYLNWYQQHPGQAPKPLIYGATNLWSGVPSRFS<br>GSGSGTDFTLTISGLQPEDFATYYCQQHGNLPFTFGTGTNLEIR
GP109	EDLLVESGGDLVQPGGSLRLSCVASGFSFSEYYMNWIRQAPGKGLEWISRIGHNGGAPKYIDSVKG<br>RFTIARDNDKNTLYLQMNSLGPDDTALYFCARGTIWVGTSHPHFDVWGAGTLVTVSS	DVQLTQPPSASASVGDTVKISCRASQSIRNYLNWYQQKPGQAPKSLIYGATNLEFGVPSRFS<br>GSGFGTDFTLTISGLQPEDFATYYCQQHNNLPFTFGAGTSLEIK
GP110	EEQLVESGGGLVQPGGSQRLSCVASGFTFNDYYMDWIRQAPGKGLEYISRIGNDGSYTTYADSVKG<br>RFTVSRDNGKNTLYLQMSGLRPEDTAVYYCVRDVHYVGGLPSFDVWGAGTLVTVSS	DVQLTQPPSVSASVGDTVQISCRASQSVGSYVNWYQQRSGQAPKPLIYYATNLQSGVASRFG<br>GSGSGTSFTLTINDLQPEDFATYYCQQHHKLPLTFGGGTRLEIK
GP111	EEQLAESGGGLVQSGGSLRLSCVASGFTFNDYYMNWIRQTPGKGLEWISRIGHNGLASKYIDSVKG<br>RFTISRDNDKNTLFLQMNNLRPEDTAIYYCTRGTIWFGTSHPYFDVWGAGTLVTVSS	DIQLTQPPSASAAVGGTVKISCRASQSVLNYLNWYRQKPGHTPEALIYGGTNLRFGVPSRFS<br>ASGSGTDFTLTISDLQPEDFAIYYCQQHKNLPFTFGAGTKLEME
GP193	EVQVLESGGGLVQPGGSLRLSCVTSGFSFTYYHLSWVRQAPGKGLEWISEVTGDSGIIEYIDSVKG<br>RFTISRDNSKNTLYLQMSSLRTEDSAVYYCAGGPLQYWGQGTLVTVSS	DVVMTQTPRLLSVSPGEPASISCRSSQSLLSSDGFTYLHWLVHKPGQAPRGVIYEVSGKYVW<br>TPERFSGSGSGTDFTLKISRVEAEDAGVYYCVQTTQYPYTFGAGTRLEIR
GP194	EEQLVESGGGLVQPGGSLKLSCVASGFIFSNSAMSWIRQAPGKGPEWISAIGRRLSNIHYADSVKG<br>RFTISRDNDKNTLYLEMSSVRPEDTAVYHCVRADDPYAFDIWGPGTLVTVSS	DVVVTQTPVLLSVSPGESASISCRSSQSLLLYDGYTYLHWLVHKPGQAPRGLIYEVSHKYSW<br>TPERITGSGSGVDFTLMISRVEAEDVGVYYCVQTTHDPLTFGGGTRLEIK
GP227	QMQLQASGPGLVEPSQTLFLTCSVSGFSITTSGYGWTWIRQPRGKTLEVMGGIAYNGSTAYNPSIR<br>SRISISRGTGKSQFSLQLNSVTEEDTGTYYCARETFSSGFLYSFFDIWGAGTLVTVSS	HILVTQTPASLAVSPGETATVNCRASQSVSKYLAWYQQKPGQAPKVLIYSASSRAIGVPARF<br>SGSGSGTDFTLTISGLQPEDVAVYYCYQYSHGGTFGGGTRLEIK
GP228	QMQLQASGPGLVKPSETLFLTCSVSGFSITTSGYGWTWIRQPRGKTLEVMGGIAYNGSVAYNPSIR<br>SRISISRDTGTNQFSLQLKSVTDEDTGTYYCTRETFSSGFTYSYFDVWGAELVTVSS	QILVTQTPASLAVSPGETGTVNCRASQSVSKYLAWYQQKPGQAPKLLIYSASSRAIGVPARF<br>SGSGSGTDFTLTISSLQPEDVAVYYCYQYSHGGTFGGGTKVEIK
GP265	EGQLVESGGGLVQPGGSLKLSCVASGFTFSAYDMNWIRQAPGKGLEWISVISSAGSTFYASSMQGR<br>FTISRDNDKNILFLQLNSLKSEDTAVYYCASGTYNFDIWGAGTLVTVSS	EIVMTQSPATLAVSPGERVTIHCKSSQSLLHSLFNKNFLNWYQQKPGRSPKLLIHLASTRAT<br>GVPDRFSGSGSGTEFTLTINNLQTEDVADYFCQQSVNAPPWTFGAGTKLEIK
GP328	EEQLVESGGGLVQPGGSLRLSCVASGFTFRNYWMNWIRQAPGKGLEWISEINRDSSTINYIDSVKG<br>RFTISRDNSKSTLYLQMSSLRSEDTAVYYCATTGGSTNYGDYYFDVWGAGTLVTVSS	SYVLTQPSSMSMSLGETARLTCEGDNIGRNYLYWYQQKPPKGPMLVMYNNKRPSGIPDRFSS<br>TKLGNTATLSITGVQAEDEADYHCQVWDSDSAHYVFGGGTKLTVL
GP374	QVQLQESGPDLVKPYQALSVTCSVSGQSITSSYSWNWIRQAPGKSLEWMGYISSGGGTGYNPSLQS<br>RISISRDTGTNQFSLQLKSITAEDTATYYCSRSGGGWGYFPLDIWGPGTLVTVSS	QPVVTQSPSASASLGAAAKLTCTLNSEYKYHNIAWFRQYPGKAPQYLMWVKSDGSLIKGSGI<br>PDRFSGSGSGTERYLTISNINSADEADYVCGSNYNIGGSFGWVFGGGTKLTVL
GP428	EAQLVESGGGLVQPGGSLRLSCVASGFTFNTYAMNWIREAPGKGLEWISAIWHDGGRMYFADSIKG<br>RFTISRDNGKNTLYLQMTNLRPEDTALYYCAKGRQYVSLFDVWGSGTLVTVSS	QPVLTQLPSASASLGQTHKLTCTLSSGYSAYNVDWYQQSQGKSPRFVMRVGPNGIVGSKGSG<br>IPDRFSGSGSGSERYLTIQNIQEEDENVYYCGADDGTGTRLVYVFGGGTKLTVL
GP435	EAQLVESGGGLVQPGGSLRLSCVASGFTFSTFAMNWIRQAPGKGLEWISMIWHDGSRMYYADSIKG<br>RFTISRDNGKNTLYLQMSSLRPEDTALYFCVKGRQYVGELDVWGAGTLVTVSS	QPVLTQLPSASASPGQTHKFTCTLNSGYSHYSVDWYQQSQGKGPRFVMRVGTTGIVGSKGDG<br>IPDRFSGSGSALDRYLTIQNIQEEDESVYYCGADYSDESSLQYVFGGGTKLTVL

Supplementary Fig. 4 | Sequence characteristics of 23 GPC trimer-binding guinea pig antibodies isolated after GPC trimer immunization.

a. ELISA assessment of 23 guinea pig antibodies.

b. Sequence characteristics of 23 binding antibodies (top) and variable domain sequences (bottom). Red font is used to highlight the neutralizing antibody gp23, with yellow and green highlighting two expanded lineages.

a

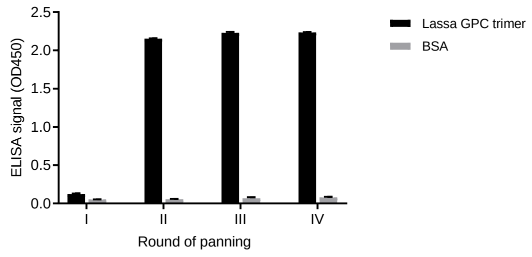

Round	Input phage (Colony forming unit, CFU)	Output phage (Colony forming unit, CFU)
I	1×10^{12}	5×10^{3}
II	1×10^{12}	5×10^{3}
III	1×10^{12}	5×10^{5}
IV	1×10^{12}	2×10^{6}

b

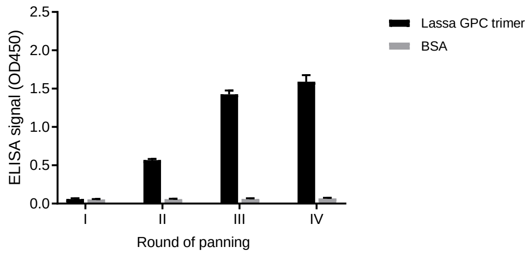

Round	Input phage (Colony forming unit, CFU)	Output phage (Colony forming unit, CFU)
I	1×10^{12}	5×10^{3}
II	1×10^{12}	3×10^{2}
III	1×10^{12}	1×10^{5}
IV	1×10^{12}	6×10^{6}

c

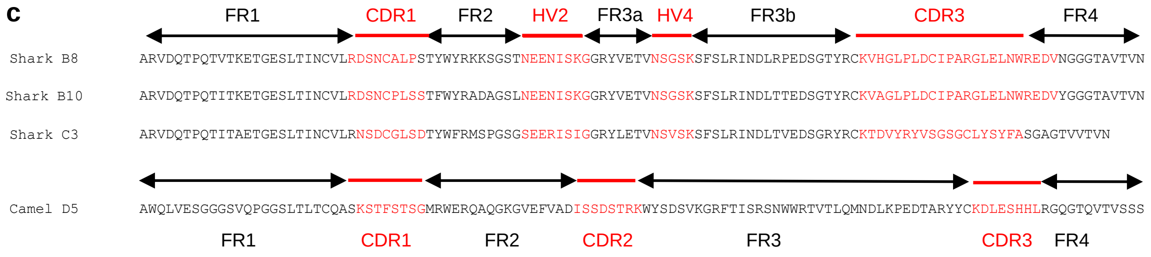

d

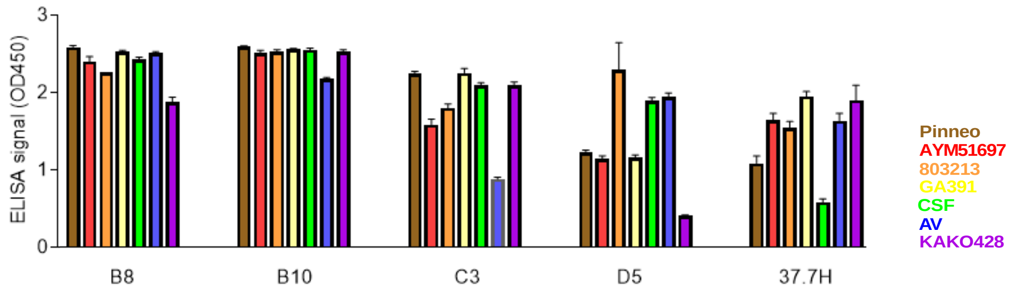

Supplementary Fig. 5 | Identification of nanobodies by panning shark VNAR and camel VHH libraries against stabilized soluble Lassa GPC trimer.

a. Phage ELISA following different rounds of stabilized Lassa GPC panning by the shark VNAR library. Input phage and output phage titers were tabulated below.
b. Phage ELISA following different rounds of stabilized Lassa GPC panning by the camel VHH library. Input phage and output phage titers were tabulated below.
c. Sequences and IMGT annotation of the nanobodies.
d. Binding of the select nanobodies toward trimers from 7 different lineages of Lassa virus.

a Lassa Josiah GPC trimer binding

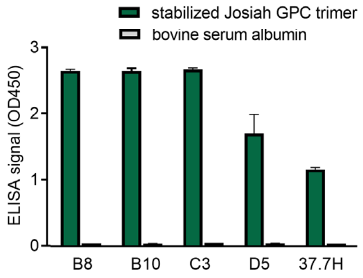

b BLI affinity measurements

Library	Antibody	Affinity (nM): stabilized Lassa GPC trimer
Shark VNAR	B8	18.8
Shark VNAR	B10	44.5
Shark VNAR	C3	19.7
Camel VHH	D5	26.7

c Cross-competition between antibodies measured by BLI

0% residual binding — 100% residual binding

Rows: Binding ligand; columns: Blocking ligand (Nanobodies: B8, B10, C3, D5; GP1-A: 10.4B, 12.1F, 19.7E; GPC-A: 8.11G, 25.10C, 36.1F; GPC-B: 18.5C, 25.6A, 37.7H)

Binding ligand		B8	B10	C3	D5	10.4B	12.1F	19.7E	8.11G	25.10C	36.1F	18.5C	25.6A	37.7H
Nanobodies	B8	16	53	55	80	92	80	79	86	83	76	72	81	90
Nanobodies	B10	65	6	77	83	89	65	91	63	93	69	78	90	77
Nanobodies	C3	83	96	18	82	55	66	70	47	38	40	86	70	73
Nanobodies	D5	90	69	88	13	89	57	70	78	79	89	21	36	35
GP1-A	10.4B	83	74	70	72	7	21	16	93	87	95	86	94	90
GP1-A	12.1F	78	81	87	80	19	5	10	88	92	96	99	91	92
GP1-A	19.7E	88	64	95	72	8	12	3	91	90	94	89	91	97
GPC-A	8.11G	87	89	58	91	87	94	90	13	10	6	79	81	92
GPC-A	25.10C	75	77	62	86	93	87	88	2	9	4	94	98	89
GPC-A	36.1F	87	92	43	93	101	98	89	4	3	11	93	88	90
GPC-B	18.5C	66	96	85	36	84	89	85	91	87	89	2	13	4
GPC-B	25.6A	77	68	80	27	88	93	95	94	96	87	15	7	2
GPC-B	37.7H	77	83	91	18	88	97	81	92	83	90	17	14	5

Supplementary Fig. 6 | Single domain antibodies identified from camel and shark library panning bind prefusion-stabilized Lassa GPC.

a. Three single domain antibodies from shark (B8, B10, C3) and one from camel (D5) libraries showed binding to the stabilized Lassa GPC trimer by ELISA. A potent human Lassa neutralizing antibody, 37.7H, was used as a positive control. Select nanobodies showed minimal reactivity toward bovine serum albumin (BSA). Triplicate measurements were made and results were represented as mean ± SEM.
b. Binding affinities of the six single domain antibodies toward the stabilized GPC trimer.
c. Cross-competition between the four single domain antibodies and human LASV-neutralizing antibodies toward the stabilized Lassa GPC trimer. Epitope binning was performed using biolayer interferometry. His-tagged stabilized Lassa GPC trimer was loaded onto the NTA sensor tips; then the blocking ligand was loaded, followed by loading of the second ligand. The numerical data indicate percent binding of the binding ligand in the presence of the blocking ligand.

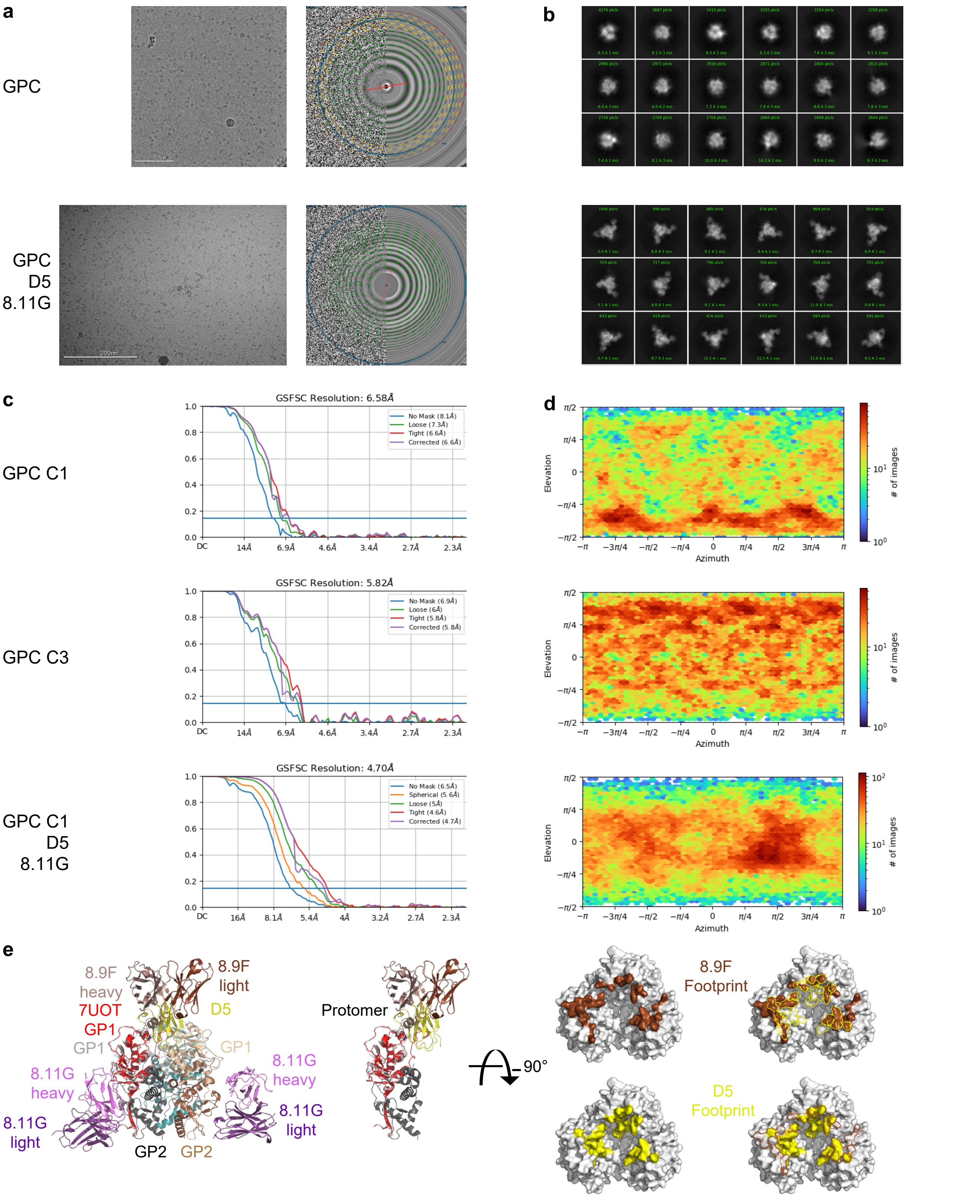

Supplementary Fig. 7 | Cryo-EM details of prefusion-stabilized GPC trimer and in complex with human Fab 8.11G and nanobody D5.

a. Representative micrographs and CTFs of the micrographs are shown.
b. Representative 2D class averages are shown.
c. The gold-standard Fourier shell correlation are shown with the resolution for the three maps.
d. The orientations of all particles used in the final refinements are shown as heatmaps.
e. Partial overlap with apex-binding 8.9F is shown, with D5 more centrally located and penetrating deeper into the apex cavity. Overlay with the outlines of the 5 Å footprint for each antibody on trimer is shown with D5 in yellow and 8.9F in brown.

Supplementary Table 1. Summary of prefusion-stabilized Lassa GPC trimers.

Construct	Stabilizing mutations	Signal peptide retained	Trimerization domain	Cleavage site	Matriglycan binding	GPC cleavage	Cell line used for expression	Yield (Trimer from 1L)	Ability to elicit neutralizing responses	Reference
**GPCysR4**	E329P, R207C-G360C	No	None	RRRR	No	Yes (cleavage site altered)	Drosophila S2 cells	Only monomer	Not reported	11
**Detergent-solubilized GPC**	None (FLAG tag for purification)	Yes	Native TM	RRLL	Yes	Yes	HEK 293F	<0.01mg	Not reported	15
**GPC-I53-50A**	E329P, R207C-G360C	No	I53-50A	RRRR	No	Yes (cleavage site altered)	HEK 293F	0.7mg	Not reported	18
**GPCysRRLL**	E329P, R207C-G360C	No	1NOG	RRLL	No	Yes	HEK 293F		Not reported	17
**GPC-cleavage intermediate**	E329P, R207GC-L326C	No	Foldon	RRRR	No	Partial (cleavage site altered)	EXPI	0.6mg	Yes (guinea pigs, low titer)	This study
**GPC-I53-50NP nanoparticle**	E329P, R207C-G360C	No	I53-50	RRRR	No	Yes (cleavage site altered)	HEK 293F		Yes (rabbit); No (guinea pig)	18
**GPC cleavage intermediate-nanoparticle**	E329P, R207GC-L326C	No	Foldon	RRRR	No	Partial (cleavage site altered)	EXPI		Yes (guinea pigs)	This study

Supplementary Table 2. BLI binding data of D5 nanobody and Fabs of human Lassa-neutralizing antibodies against prefusion-stabilized LASV GPC trimer.

Nanobody		GPC
B8	K_D (M)	1.88 E-08
	k_{on} ($M^{-1}s^{-1}$)	1.76 E+05
	k_{dis} (s^{-1})	3.31 E-03
B10	K_D (M)	4.45 E-08
	k_{on} ($M^{-1}s^{-1}$)	2.23 E+05
	k_{dis} (s^{-1})	9.92 E-03
C3	K_D (M)	1.97 E-08
	k_{on} ($M^{-1}s^{-1}$)	3.49 E+05
	k_{dis} (s^{-1})	6.88 E-03
D5	K_D (M)	2.67 E-08
	k_{on} ($M^{-1}s^{-1}$)	1.21 E+05
	k_{dis} (s^{-1})	3.23 E-03

Fab		
10.4B	K_D (M)	6.63 E-08
	k_{on} ($M^{-1}s^{-1}$)	5.38 E+04
	k_{dis} (s^{-1})	3.57 E-03
12.1F	K_D (M)	3.64 E-08
	k_{on} ($M^{-1}s^{-1}$)	2.81 E+04
	k_{dis} (s^{-1})	1.02 E-03
19.7E	K_D (M)	1.57 E-07
	k_{on} ($M^{-1}s^{-1}$)	8.48 E+03
	k_{dis} (s^{-1})	1.33 E-03
8.11G	K_D (M)	3.71 E-08
	k_{on} ($M^{-1}s^{-1}$)	3.68 E+04
	k_{dis} (s^{-1})	1.37 E-03
25.10C	K_D (M)	8.56 E-09
	k_{on} ($M^{-1}s^{-1}$)	2.82 E+05
	k_{dis} (s^{-1})	2.41 E-03

Fab		
36.1F	K_D (M)	1.52 E-08
	k_{on} ($M^{-1}s^{-1}$)	1.71 E+05
	k_{dis} (s^{-1})	2.61 E-03
18.5C	K_D (M)	7.31 E-09
	k_{on} ($M^{-1}s^{-1}$)	5.35 E+04
	k_{dis} (s^{-1})	3.91 E-04
25.6A	K_D (M)	1.54 E-08
	k_{on} ($M^{-1}s^{-1}$)	1.64 E+05
	k_{dis} (s^{-1})	2.53 E-03
37.7H	K_D (M)	4.47 E-09
	k_{on} ($M^{-1}s^{-1}$)	1.85 E+05
	k_{dis} (s^{-1})	8.28 E-04
