# Supplementary material for: Cleavage-intermediate Lassa virus trimer elicits neutralizing responses, identifies neutralizing nanobodies, and reveals an apex-situated site-of-vulnerability
Source: Nat Commun. 2024 Jan 4;15:285. doi: 10.1038/s41467-023-44534-y (PMC10767048; doi:10.1038/s41467-023-44534-y)
Supplement: Supplementary file 3 — Description of Additional Supplementary Files [file 41467_2023_44534_MOESM3_ESM.docx]

**Description of Additional Supplementary Files**

**Title**: Supplementary Data 1 | Design rationales and sequences of 164 Lassa GPC trimer constructs.

**Description**: This excel file provide explicit amino-acid sequences for 164 Lassa GPC trimer designs, color-coded by their design rationale.
